# Supplementary material for: Comparison of sampling methods for small oxbow wetland fish communities
Source: PLoS One. 2022 Nov 17;17(11):e0277698. doi: 10.1371/journal.pone.0277698 (PMC9671342; doi:10.1371/journal.pone.0277698)
Supplement: Attachment — Submitted filename: Response to Reviewers.docx [file pone.0277698.s002.docx]

**Reviewer 1 Comments**-

The manuscript PONE-D-22-06964 entitled ,,Comparison of sampling
methods for small oxbow wetland fish communities'' deals with four
sampling methodologies in order to find an optimal gear to sample fish
communities in small oxbow wetlands.

The Authors sampled 12 small oxbows with fyke netting, seining,
backpack electrofishing and minnow traps. Each oxbow was sampled one
time with each gears. Although the oxbows were small, their sizes
varied considerably (the shortest was 23 m and the longest was 139 m
in length). There were three study variables used to compare the four
methods: 1) catch per unit effort (CPUE), 2) species richness, and 3)
10th-90th quantile range in total length of the fish. They used mixed
effect linear models for each study variables to make statistical
inferences on the differences between the four gear types.

Authors found significant differences between the sampling gears in
all three study variables. After considering the results and
discussing the benefits and drawbacks of the use of each gear type,
the Authors concluded that minnow trapping is ineffective to sample
fish communities in oxbows, but seining and fyke netting can be an
optimal method.

In my opinion, the manuscript (MS) is very well written and
readable. The evaluation and discussion of the use of the studied
sampling gears in the field are correct and can be useful for field
managers if they want to choose methods to sample fish communities. On
the other hand, the data analysis approach they used seems not to be
appropriate to reach a conclusion on 'optimalness' of the studied
gears.

**We have reworded instances in the manuscript where the term “optimal” was used as this is not what is truly being tested here. The goal of our study was to determine what survey methods are effective (as defined in the MS, line 94) for sampling oxbow fishes.**

Specifically, the Authors computed CPUE as the number of fish caught
divided by 100 m2 oxbow surface area. They chose this way to compute
the CPUE, because they ,,sought to compare the performance on each
gear type in terms of ability to sample the entire fish community
within an oxbow''. My main concern is that the four types of sampling
gear can not be compared in this way, because none of them is supposed
to yield representative picture on the true (entire) fish community of
the oxbow (effectiveness of each method is effort-dependent). In
addition, seining was applied along the entire length of the oxbow
(L150), backpack electrofishing was conducted by walking along the
entire shoreline perimeter (L134). That is, sampling effort was
proportional to the size of the oxbows in case of these two
gears. Contrary, three fyke nets (L138) and four minnow traps (L145)
were exposed in each oxbows. In other words, fyke netting and minnow
trapping were used with constant effort, regardless of the size of the
oxbows. Because size of the sampled oxbows varied considerably,
effectiveness of the fyke netting and minnow trapping certainly varied
as well. Consequently, the catch result of the fyke netting and that
of the minnow trapping should only be considered as conditional data
obtained with a given number of traps (three and four) and exposure
time. Therefore, dividing the catch data by 100 m2 oxbow surface can
not make the data comparable to each other. This division is not a
proper way to compute CPUE for passive gears (e.g., fyke nets),
because the real effort was constant at each oxbow. Relative
effectiveness of the gears can not be judged, because the sampling
effort was proportional to the oxbow size in case of seining and
electrofishing, but it was not proportional to that in case of the fyke
netting and minnow trapping. So, the application of the CPUE computed
in the way as the Authors did, appears to be senseless to me, I am
afraid. Meaningful CPUE for each gear would be something like this:
number of fish individuals caught per X m walking (or sampled area)
for electrofishing, number of fish individuals caught per X m2 sampled
area for seining, number of fish individuals caught per one trap per
hour for the fyke nets and minnow traps.

**We agree with the reviewer that our use of CPUE and comparisons between sampling methods can be improved. Therefore, instead of lumping all four sampling methods together, we have split these sampling methods into active (seining and electrofishing) and passive (fyke netting and minnow trapping) and have added language to indicate and discuss the possibility of under-sampling by using constant effort for passive methods. CPUE in the way that we calculated it is perhaps most problematic for minnow trapping (as we have noted in the MS). However, as we have discussed in the MS, it is unlikely that increasing the number of minnow traps deployed, or calculating CPUE in terms of the amount of time each trap was present within the oxbow, is unlikely to improve the performance of this gear type. Many studies (cited in the MS) have found minnow trapping to be an ineffective sampling method to document species richness. Furthermore, other studies have noted that minnow trapping is inherently size selective given the small opening size for minnow traps. For seining and backpack electrofishing, we do understand the reviewers disagreement with our calculation of CPUE, however, we contend that the method we employed is appropriate for this study. We believe this as the goal of our study was to determine how these sampling methods performed in sampling the entirety of the oxbow, not just the area that the gear was able to effectively sample. For instance, if we calculated CPUE for electrofishing using the amount of area sampled, these results of electrofishing would actually be inflated as the amount of habitat that was unsampled would not be included in the CPUE calculation. This unsampled habitat is critical to include given that we considered each oxbow as one closed unit and were interested in comparing gears in terms of how they sampled the entirety of this unit.**

**Finally, our method for calculating CPUE allows for direct comparisons between fyke netting (a passive gear) and seining/electrofishing (active gears) which provided interesting results. We determined that fyke netting performed equally as well as seining, despite the fact that we used constant effort across site sizes and did not scale the number of nets to oxbow size. If fyke netting CPUE was calculated based on area sampled (which is unknown), or on time of deployment, we would not be able to effectively make these comparisons.**

Therefore, I would suggest an alternative way to analyze the
data. Pool the species list obtained with the different gears for a
given oxbow. Consider this as a proxy of the true species pool (fish
species living in the oxbow). Compute pairwise ecological
dissimilarities (or similarities) in species composition (i.e., list
of species detected) between each gear and the estimated true species
pool. Test the gears for differences in the degree of dissimilarity to
the estimated species pool if you would like to. Because conservation
management is often interested in detection of species presence, it
could be an interesting part of your analysis, if species caught with
all three methods, and species caught with only one gear type were
identified (if there were such species at all).

**We agree with the reviewer that the species richness data is perhaps the most interesting finding of our study. Therefore, we have included this additional analysis in the revised manuscript which further supports our conclusions.**

Despite the correct discussion and the probably plausible conclusion
the Authors reached, I think the results may be misleading due the
issue of the data analysis explained above. Therefore, I recommend the
reanalysis of the data and reworking the paper according to the new
result. After doing so, the paper should be resubmitted for
publication. Please find my major issues with references to the lines
of the paper below. In addition, there are some minor issues that
should be checked and corrected too (please see below).

**We have added language to address why we calculated CPUE in the way that we did and to indicate that future studies should examine scaling the number of traps or fyke nets to oxbow size. We appreciate the reviewers constructive comments and believe our manuscript to be improved as a result.**


* Major issues and Comments
----------------------------------
L94 You consider a method 'optimal' if it provides accurate
information for abundance. What do you mean by abundance? Total number
of fish living in the oxbow. If so, I think the use of constant number
of fyke nets/traps ab ovo gave biased data. As far as I know, it is
very difficult, if possible at all, to estimate accurately the total
population size, especially with single pass electrofishing and
seining, not to mention the passive gears.
**Reworded the sentence to clarify that an effective method would produce consistently high catch rates as we are not trying to estimate total population size.**

L134 and L150 Electrofishing along the entire shoreline involves
sampling effort proportional to the size of oxbow, just as seining
along the entire length of the oxbow. (see the next comment)

L138 and L145 Constant number of fyke nets and minnow traps, however,
gave data disproportionate to the size of the oxbow. So, the catch of
the electrofishing and seining can be considered as more
representative in terms of the given gear type, than the catch of the
fyke nets and minnow traps.
**It is possible that under or over sampling of the habitat occurred when using fyke nets or minnow traps. However, it is unlikely that scaling the number of minnow traps to oxbow size would improve performance given the inherent sampling biases of these gears. Additionally, fyke netting performed very similarly to seining in terms of sampling the fish community of the oxbow sites.**

L158-164 I am afraid I do not agree with this arguing. The catch of
the gears can be standardized only in a gear specific way. If you want
to use the oxbow surface area, I can imagine a way like this how to do
that: you can compute a ratio of the sampled area to the entire
surface area of the oxbow and that could be a rough indicator of the
effort level used to obtain information on true fish community in a given
small oxbow. But the effective zone of the fyke nets and minnow traps
are unknown which make impossible to estimate a similar effort level
for these gears. So, I can not see a proper way to standardize your
data across the gears.
**As mentioned previously, the unsampled area is important for us to include in our CPUE metric as the goal of our study was to compare how each gear sampled the entirety of the oxbow. If this value was not included, catch for electrofishing would be inflated using the method the reviewer presents here as unsampled area (deep water) would be treated as sampled area, even though electrofishing cannot possibly sample the fish in that area. Given the goals of our study, we believe that this is the most appropriate way to calculate CPUE.**

L173 I have not found the numerical results of the models in the MS.

**These have been added within the results section.**

L176 If I understand correctly your design, a factor with four levels
corresponding to the gears, and a discrete covariate, I presume the
week of the year when the sampling was conducted, were used as
independent variables in your models. The interaction between the
factor and the covariate was not included. The sample size for each
level of the factor were the same, so you had a balanced design. Why
did you use type III ANOVA? As far as I know, in case of having no
interaction, type II ANOVA is more powerful than the type III
one. Although, both should be expected to give the same result when
the design is balanced, so probably you would have got the same result
with the type II tests as well. Yet, it would be better to use the
type II test due to you are not interested in the interaction, or you
find the interaction term insignificant.

**We have rerun analyses using a type II test, which as the reviewer noted, did not change model results.**

L199-201 In my opinion, this kind of results would be the most
interesting for conservation managers. It would also worth to analyze the
species composition (species detected/not detected) for each gear.

**We agree that the species richness findings are most interesting to managers and have increased our discussion of them in the MS, including adding a new fish community metric as recommended by the reviewer.**

L211 I have not found reference to the species accumulation curves in
the Methods section. Why did you make this plot (Fig2)? As I see, it
informs on how the number of species detected accumulates with the
number of oxbows sampled. Although it could be a good illustration of
the overall effectivity of the gears, but it must keep in mind that only one
sampling was carried out for each oxbow with each gear type with
sampling effort variyng across the four gears. Moreover, the
composition of the fish communites in the oxbows (i.e., the
statistical populations) varied obviously too. Steepness of species
accumulation curves depends on the true species richness. Finally, if
repeated sampling were applied, this curve would look different.
(Anyway, a highly valuable part of the study would be, if you select
randomly 5-6 oxbows and make an effort analysis for each gear. I mean
how the number of species detected accumulates as a function of the
effort (sampled length, number of traps etc.) within a single
oxbow. This curve averaged across the 5-6 oxbows for each gear would
be informative on the efficiency of the gears for your oxbows.)

**As repeated sampling was not applied in this study, the species accumulation curves as presented were not appropriate. Therefore, we have removed these curves and any reference to them.**

L248-249 This is a self-evident result. The opening of the minnow
traps was originally small in diameter (2.5 cm, L145).
**Yes, but is still important to note as these gears have been used by other studies attempting to document fish communities.**

L269-271 This is a weak point of the study which basically aims to
compare sampling methods, I think.
**This study aims to compare how each sampling method documented species richness across sites, performed in terms of total catch and sampling bias. Our goal was not to compare the efficiency of gear types within these sites.**

L272-274 I am afraid I can not accept this as a supporting
explanation. Strong relationship between two or more measures, e.g.,
species richness and habitat variables, does not imply effect size or
representativeness. The goal of your study is comparison of data
obtained with different gears. Effect size (differences between means)
and compositional differences matter here. Whereas studies on
fish-habitat relationships primarily aim to identify variables and
factors which describes the variation of the biota, and estimate their
relative importance in explanatory power.
**We agree with the reviewers comment and have removed this reference.**

L274-276 In my opinion, the finding regarding to the species richness
is the most important result of your study. This should be emphasized
in the reworked MS.
**We agree with the reviewers comment and appreciate their constructive edits for this manuscript.**

* Minor issues and Comments
----------------------------------
L110 What do you mean by centerline of the oxbow? Length measured
along the midpoints of transects laid equally spaced on the oxbow?

**Added clarification**

L165 Do you mean that the 10th-90th quantile range were computed for
the catch of each gear?

**Yes, and only one gear was sued for each sampling event.**

L175 What do you mean by 'week'? Perhaps the week of the year?

**Week of the sampling period, added to clarify**

L177 Emmeans function of the R?? Which package from? (I suppose the
emmeans package.)
**Packages used have been listed in methods.**

L180 Maybe it would be correct if this sentence were rewritten as
something like this: 'Result of a comparison were considered as
statistically significant if the P-value of the test was less than or
equal to 0.05.' (My problem is that the critical value or threshold to
make a statistical inference is a single figure, usually 0.05, and not
an interval. This is the significance level of the statistical
inference (the alpha value). But the p-value obtained for a certain
test is the significance value of the test under question.)

**Rewritten as the reviewer indicated.**

L253 I suppose the 24-26 species regards for all oxbows? Could you
clarify that?

**Added to clarify**

Reviewer #2: My recommendation is to reject the manuscript in its present state. The manuscript does not present quantitative or qualitative data for 3 of 4 objectives. The fish metrics were quantitively presented and are a valuable contribution to the literature. I have included recommendations to potentially address two additional objectives. I do not believe that in the absence of habitat measurements that the habitat disturbance objective can be addressed and should be removed.

The data presented in Figure 3 strongly suggest that the assumptions of the linear mixed model should be checked.

**Assumptions were checked as described in methods and data was transformed as necessary.**

**Reviewer 2 Comments**-

These data are very publishable, and I encourage the authors to read this review carefully. This was written with the goal of seeing these data published. The manuscript needs to remove and greatly revise discussion of stress to fish, habitat disturbance, and ease of use. These objectives were neither qualitatively or quantitatively measured (e.g., time for deployment in terms of person/hours, percentage of disturbance to substrate and vegetation per unit area) nor were available literature used to support the assertions (i.e., fish stress following electrofishing versus net-based methods is well documented). Much of the discussion on stress, habitat disturbance, and ease of use is subjective and speculative. Either address by providing data, use the literature, or restrict the manuscript to quantitative comparisons of the fish assemblage and metrics. These comparisons are valuable and are publishable. Below, I have recommendations for revision. Some of these are substantial. CPUE and richness are often not normally distributed and usually not linear. Please describe how you determined that a linear mixed model was appropriate. In the Results, statistical outcomes should include the test statistic and degrees of freedom along with pvalues. P-values themselves (so called naked p-values) lack important information. A better Results section would have “Total CPUE was significantly greater (F = X, p < 0.001) for fyke netting (CPUE = X1, Y1 standard error) and seining (CPUE = X2, Y2 standard error) than backpack electrofishing (CPUE = X3, Y3 standard error ) and minnow trapping (CPUE = X4, Y4 standard error)” and similar statements for length and richness. If you did this, Figure 1 would be helpful as presented, although the version described below would still be better. Without the aforementioned Results, Figure 1 is not as helpful as presented. A better version would present the CPUE, richness, and length ranges as y-axis with box plots by method. Comparisons would be better depicted by lines and letters or by a separate table (better). The mean CPUE, richness, and length per method allows the reader to understand how you arrived at your conclusions and how to adapt your results to their situation. For example, someone may not have access to a fyke net, but has access to seines and minnow traps. They could then have some expectation of the I strongly recommend that if you still have the data, present the actual time spent with each method in person/hours. In the current manuscript, whereas you did not quantify effort in terms of time spent sampling, it is difficult to address the time versus data quality of data trade off. One reason that electrofishing is popular is that in a relatively short period of time (e.g., drive there, sample three 15 minute passes plus identification and processing, drive back), you have a completed sample. You did an entire perimeter, and without the time spent reported presented in the manuscript (probably longer than 45 minutes), it is difficult to determine how long you spent electrofishing. However, it certainly was less time than driving out, deploying fyke nets, driving back, driving there again, retrieving nets, identifying and processing fish, and driving back. Without time spent data, you could make a good argument that active methods like seining and electrofishing take about the same amount of time and seining produced better data in your oxbows. You cannot really compare the two passive methods to the active methods in the same way. The manuscript also could be enhanced by using the literature to address fish stress. You could make a table presenting literature comparisons among methods to support your assertations about handling stress and mortality associated with each method. Then, you could use these comparisons to qualitatively compare the methods (i.e., fyke nets had lowest stress and second lowest mortality of compared methods, whereas seining had the second lowest stress and lowest mortality. Both of these methods had much lower stress than electrofishing). It is not clear that the habitat disturbance objective is comparable. Simply assuming that electrofishing, but not seining, tramples habitat in a meaningful way without some measure of the habitat is speculative. I could argue that seining, by its contact with vegetation and substrate, is far more destructive than walking around. This is the weakest objective and probably just needs to be removed. I did not make line-by-line comments, given the revisions requested. The writing was quite good, and I would not expect to make many line-by-line comments.

**We appreciate this reviewers constructive comments and noted wanting to see this data published. As recommended by the reviewer, we have removed the majority of discussion of habitat disturbance and ease of use, reducing these discussion to just a few sentences. Furthermore, we have eliminated our mention of the “qualitative” metrics for comparison and have instead provided brief commentary pertaining to these metrics (ease of use, potential stress to fish, potential habitat disturbance). Also, we limited this discussion to our top performing gears (seining and fyke netting). In terms of stress and habitat disturbance, we have added citations were appropriate to indicate how fyke netting or seining my stress fish or cause habitat disturbance. We have rewritten the results section as recommended by this reviewer. Additionally, we have reconstructed figure 1 as described by the reviewer. We agree with the reviewer that effort (total time spent sampling, driving, maintaining gear etc) data would be beneficial to this manuscript, however, this data was not collected. We searched literature to find studies examining fish stress relating to seining and fyke netting, however, our search turned up little relevant research. Therefore, our discussion of fish stress was reduced but appropriate citations were included were relevant.**
