# Supplementary material for: Comparison of sampling methods for small oxbow wetland fish communities
Source: PLoS One. 2022 Nov 17;17(11):e0277698. doi: 10.1371/journal.pone.0277698 (PMC9671342; doi:10.1371/journal.pone.0277698)
Supplement: Attachment — Submitted filename: Response to Reviewers.docx [file pone.0277698.s003.docx]

Journal Requirements:

When submitting your revision, we need you to address these additional requirements.

1. Please ensure that your manuscript meets PLOS ONE's style requirements, including those for file naming. The PLOS ONE style templates can be found at
   https://journals.plos.org/plosone/s/file?id=wjVg/PLOSOne_formatting_sample_main_body.pdf and 
   [**https://journals.plos.org/plosone/s/file?id=ba62/PLOSOne_formatting_sample_title_authors_affiliations.pdf**](https://journals.plos.org/plosone/s/file?id=ba62/PLOSOne_formatting_sample_title_authors_affiliations.pdf)**.**

**All requirements have been checked and corrections have been made where necessary.**

2. Thank you for stating the following in the Acknowledgments Section of your manuscript: 
"We thank our field technicians, S. Grinstead and C. Wood, for the many hours spent in the field collecting the data presented within this manuscript. We also thank the various collaborating entities and individuals who have made this project possible, either through funding or support of the research efforts. Collaborators for this project include C. McKinney, K. Wilke, A. Kenney, J. Olson, D. Weissenfluh, Iowa Soybean Association, Syngenta Crop Protection LLC, The Nature Conservancy, and the U.S. Fish and Wildlife Service. Finally, we thank the many landowners whose support was vital for the success of this project".

We note that you have provided funding information that is not currently declared in your Funding Statement. However, funding information should not appear in the Acknowledgments section or other areas of your manuscript. We will only publish funding information present in the Funding Statement section of the online submission form. 
Please remove any funding-related text from the manuscript and let us know how you would like to update your Funding Statement. Currently, your Funding Statement reads as follows: 
 "The author(s) received no specific funding for this work."

Please include your amended statements within your cover letter; we will change the online submission form on your behalf.

**Amended funding statement has been included in the cover letter and all financial mentions have been removed from the manuscript.**

3. We note that you have stated that you will provide repository information for your data at acceptance. Should your manuscript be accepted for publication, we will hold it until you provide the relevant accession numbers or DOIs necessary to access your data. If you wish to make changes to your Data Availability statement, please describe these changes in your cover letter and we will update your Data Availability statement to reflect the information you provide.

4. Please note that in order to use the direct billing option the corresponding author must be affiliated with the chosen institute. Please either amend your manuscript to change the affiliation or corresponding author, or email us at plosone@plos.org with a request to remove this option.

**Corresponding author is now Timothy Stewart (twstewar@iastate.edu)**

5. Please include your full ethics statement in the ‘Methods’ section of your manuscript file. In your statement, please include the full name of the IRB or ethics committee who approved or waived your study, as well as whether or not you obtained informed written or verbal consent. If consent was waived for your study, please include this information in your statement as well.

**Requested statement and IACUC permit information has been added within the methods section.**

6. Please review your reference list to ensure that it is complete and correct. If you have cited papers that have been retracted, please include the rationale for doing so in the manuscript text, or remove these references and replace them with relevant current references. Any changes to the reference list should be mentioned in the rebuttal letter that accompanies your revised manuscript. If you need to cite a retracted article, indicate the article’s retracted status in the References list and also include a citation and full reference for the retraction notice. Additional Editor Comments:
**References have been reviewed and an error was found with reference #30 being placed prior to #27, this has been fixed. Additionally, a reference was added to an article recently published pertaining to oxbow restoration monitoring and Topeka Shiner recovery which is highly relevant to this study (Osterhaus et al. 2022).**

Dear Dr Osterhaus,

I have now received comments from the two reviewers that assessed a previous version of the manuscript.
Both reviewers agreed that the revised version is greatly improved, and I agree with this view.
Before I am able to accept the manuscript for publication, you need to address a key issue raised by rev.1. This is regarding the definition of CPUE. For coherence, the reviewer proposes an update of the definition used in your study, to account of the different gears used.
Please consider the suggestions and modify the text accordingly, to the extent that you consider agreeable.

I therefore recommend minor revision at the moment.

Looking forward to receive the latest version.

**All suggestions made by reviewers have been addressed at this time. We thank the editor for their comments and guidance during this process.**

**Comments to the Author**

Reviewer #1: * GENERAL COMMENTS
The study reported in the manuscript PONE-D-22-18618 ("Comparison of
sampling methods for small oxbow wetland fish communities") compared
four sampling methodologies, two active and two passive gears, with
regard of effectiveness in sampling fish communities of small wetland
oxbows.

I have reviewed a former draft of the MS. The Authors have reworked
the paper, and they have considered and applied the suggestions of the
former reviewers. As a result, the MS has improved a lot. It is a well
written paper, contains relevant results and suggestions for
monitoring fish communities in small oxbows. I would like to highlight
the merit of the Discussion, which has valuable parts regarding the
practical use of the each gear in the field.

However, one issue have remained in the text that I do not agree
with. See my SPECIFIC COMMENTS below for the details, where I have
made a suggestion (an easy conceptual correction) to solve that issue.
Please consider my suggestion or another change in order to make your
paper more clear and exact.

**The authors greatly appreciated the constructive comments and recommendations provided in the first round of review and agree that the manuscript has been greatly improved as a result of these comments and recommendations.**

In my opinion, the MS is publishable provided that the authors correct
the issue in question. I have some small notes as well that need to be
revised; please refer to the SPECIFIC COMMENTS.

* SPECIFIC COMMENTS
L140-141 Please correct the notes for the unit of measure of
conductivity. The correct form has a capital "S", which stands for
"Siemens".

**The S has been capitalized in each usage.**

L151-152 The sentence starting "Future studies..." maybe would be in a
better place in the Discussion. But, I see reason why you have written
that here. So, it can also remain here, but it makes a small
"stylistic" dissonance because this sentence evaluates.

**We agree that this sentence creates minor dissonance within the paragraph, however, we believe that the best fit for this sentence is in the location that it is currently placed. We attempted to find a better location within the discussion but could not find a placement that improved flow.**

L166-172 This is the issue that I do not agree with. As far as I am
concerned, Catch Per Unit Effort (CPUE) is an applicable way to
standardize data sampled only with the *same* sampling
methodology. The sampling effort is basically gear-specific (e.g.,
electrofishing time, exposure time, the number of fyke nets used, and
so on), so it can not be comparable between gears. Consequently, your
CPUE ("the number of fish collected per 100m2 of oxbow surface area")
as it is now, is not a real CPUE. On the other hand, your data need to
be standardized to control for the size differences of the
oxbows. Hence, I understand the reason why you scaled your raw catch
data to unit surface area; it is correct. But to be precise in the use
of concepts, I do recommend changing the response variable CPUE to
"catch per unit surface area" (CPUS) or "catch per unit area" (CPUA),
and leaving the CPUE. In summary, if I were you, I would highlight the
need for scaling the raw data to control for the size differences of
the oxbows, instead of reasoning with "the ability to sample the
entire fish community within an oxbow". In fact, dividing by the area
does not provide information on the entire fish community, so I am
afraid I cannot accept your argument. Please reconsider this part of
your paper. I think that the small correction I suggested would make
the text more accurate and precise.

**This is an excellent point and very well laid out. We agree with the reviewer that CPUE is not appropriate and is really not what were calculating. Therefore, we have made edits to the manuscript and figures where needed to change from CPUE to CPUA which is a much more relevant definition and accurate to what we were calculating.**

L189 Maybe the word "levels" is not necessary because readers that are
less familiar with linear models or the jargon of statistics may not
understand what "levels" mean. You sampled each oxbow only once, so
your factor 'oxbow' in the models have 11 levels each with one
repetition. Therefore, averaging over the levels eventually means
averaging over the oxbows, if I am not mistaken. An other option can
be giving a little bit more explanation on what "levels" means.

**We agree with the reviewer that the usage of “levels” here is unnecessary and have made the appropriate change.**

L 320-321 Please consider changing the word "sites" to "oxbows".

**Site and Sites have been changed to Oxbow and Oxbows**

Reviewer #2: I reviewed an earlier version of this manuscript and made the following over-arching recommendations, as well as some more minor recommendations. Given the revisions, I recommend acceptance.
“The manuscript needs to remove and greatly revise discussion of stress to fish, habitat disturbance, and ease of use. These objectives were neither qualitatively or quantitatively measured (e.g., time for deployment in terms of person/hours, percentage of disturbance to substrate and vegetation per unit area) nor were available literature used to support the assertions (i.e., fish stress following electrofishing versus net-based methods is well documented). Much of the discussion on stress, habitat disturbance, and ease of use is subjective and speculative. Either address by providing data, use the literature, or restrict the manuscript to quantitative comparisons of the fish assemblage and metrics. These comparisons are valuable and are publishable. Below, I have recommendations for revision. Some of these are substantial.”
The authors discussion of ease of implementation, interactions with habitat, and apparent mortality/stress are greatly improved. Number of visits and hours spent per visit are very important considerations, as is disturbance during sensitive life history stages.
The authors also did address minor recommendations. For example, the nonlinearity, non normality of the fish data were addressed, test statistics were added, and figure 1 was improved.

**We thank the reviewer for their constructive feedback and appreciate their time and effort.**
